# Association of the comorbidity of gestational diabetes mellitus and hypertension disorders of pregnancy with birth outcomes

**DOI:** 10.3389/fendo.2024.1468820

**Published:** 2024-12-12

**Authors:** Xingxi Lin, Luhan Zhou, Shuting Si, Haoyue Cheng, Xialidan Alifu, Yiwen Qiu, Yan Zhuang, Ye Huang, Libi Zhang, Diliyaer Ainiwan, Hui Liu, Yunxian Yu

**Affiliations:** ^1^ Department of Public Health, and Department of Anesthesiology, Second Affiliated Hospital of Zhejiang University School of Medicine, Hangzhou, China; ^2^ Department of Epidemiology and Health Statistics, School of Public Health, School of Medicine, Zhejiang University, Hangzhou, China; ^3^ Department of Health Care, Yiwu Maternity and Children Hospital (Yiwu Branch of Children’s Hospital Zhejiang University School of Medicine), Yiwu, China; ^4^ Sir Run Run Shaw Hospital, School of Medicine, Zhejiang University, Hangzhou, China

**Keywords:** gestational diabetes mellitus, hypertension disorders, pregnancy, adverse neonatal outcomes, comorbidity

## Abstract

**Backgrounds:**

Many pregnant women suffer from more than one pregnancy complication. However, whether those women experienced a higher risk of adverse birth outcomes is unclear. This study aims to assess the association between the comorbidity of gestational diabetes mellitus (GDM) and hypertension disorders of pregnancy (HDP) and adverse birth outcomes.

**Methods:**

The data was from the Zhoushan Maternal and Child Health Hospital electronic medical recorder system (EMRS) between 2015 and 2022. Multivariate linear regression model was used to analyze the association of GDM, HDP, and comorbidity with birth weight and gestational age, respectively. Multiple logistic regression model was used to analyze the association of GDM, HDP, and comorbidity with adverse birth outcomes.

**Results:**

13645 pregnant women were included. GDM+HDP was significantly associated with a higher risk of composite adverse neonatal outcomes (OR=1.82, 95%CI: 1.02-3.04), including preterm birth, placenta previa, and/or neonatal jaundice, a higher risk of small for gestational age (SGA) (OR=2.2, 95% CI: 1.24 3.92) and large for gestational age (LGA) (OR=2.33, 95% CI: 1.64 3.31) compared with the normal group. Further analysis showed that HDP diagnosed in the 21-27^th^ week comorbid with GDM had the lowest gestational age at delivery (β= -1.57, *P*=0.0002) and birth weight (β= -189.57, *P*=0.0138). Moreover, combined hyperglycemia (CH) comorbid with HDP had the strongest association with reduced gestational age (β= -0.83, *P*=0.0021).

**Conclusion:**

Pregnant women suffering from both GDM and HDP had a higher risk of adverse neonatal outcomes; hence, the prevent and treatment of GDM and HDP, especially their comorbidity, are very important for pregnant women.

## 1 Introduction

Gestational diabetes mellitus (GDM) is defined as a new onset or first recognition of glucose intolerance during pregnancy. According to a report, 12.8% of pregnant women suffered from GDM worldwide and the incidence of GDM has reached 14.8% in China, with an increasing growth trend (1). Hypertensive disorders in pregnancy (HDP) are a group of maternal disorders characterized by elevated blood pressure during pregnancy, including gestational hypertension, preeclampsia, and eclampsia. The global prevalence of HDP has been reported to range from 4.6% to 13.1% (2), and among Chinese pregnant women, it is approximately 5% to 10% (3). Both GDM and HDP are associated with a risk of adverse birth outcomes, including newborn birth weight, preterm birth (PTB), placenta praevia, premature rupture of membranes, and placental abruption. Long-term complications of GDM include obesity, diabetes, and cardiovascular disease in the mother and offspring. HDP increases the risk of future coronary artery disease and chronic kidney disease.

Both GDM and HDP are among the most common complications of pregnancy. Recently, the prevalence of GDM and HDP has increased rapidly. Pregnant women with both diseases pose a great challenge for clinical management. Previous studies have shown that GDM and HDP were closely related, and women with GDM were at a significantly increased risk of hypertension and preeclampsia (4). The co-morbidity of GDM and HDP may further increase the risk of adverse birth outcomes. However previous studies have mostly investigated the effect of having only one of these diseases on adverse outcomes. Few studies have been conducted on the co-morbidity of GDM and HDP, whose interaction is unclear.

The relationship between a single condition of GDM or HDP and adverse outcomes has been well established. GDM was associated with adverse outcomes such as macrosomia, pre-eclampsia, low birth weight, birth trauma (shoulder dystocia), respiratory distress, cesarean delivery, neonatal intensive care unit (NICU), and fetal death (5, 6). HDP increased the risk of preterm birth, stillbirth, small for gestational age (SGA), and low birth weight (3, 7). PE significantly increased the risk of placental abruption (8). Studies have shown that in pregnant women with GDM combined with PE, excess gestational weight gain (GWG) led to a more pronounced increase in the risk of preterm delivery and the occurrence of older than gestational age (LGA) infants (9), and the severity of their PE was positively associated with SGA (10), suggesting that co-morbidity of GDM HDP may have a significant impact on adverse birth outcomes. Another study showed that diabetes mellitus combined with hypertension significantly increased the incidence of PTB, but in this study, it was chronic diabetes mellitus rather than GDM (11). A UK study showed that GDM combined with gestational hypertension significantly increased the incidence of LGA and cesarean delivery (6). Another Taiwanese data showed that women with HDP had a higher risk of neonatal PTB and SGA and women with both HDP and GDM had a further increased risk of PTB, SGA, and LGA, but this study did not compare the co-morbidity of GDM and HDP with GDM alone (12).

In summary, only a few studies have explored the effect of GDM and HDP co-morbidity on some adverse birth outcomes, but the relationship between GDM combined with HDP and multiple adverse birth outcomes has not been elucidated. And they didn’t consider that diagnosis of HDP at different periods and GDM subtypes may have different effects on outcomes. Besides, few relevant studies have been conducted in mainland China. Therefore, studies on the co-morbidity of GDM and HDP are inherently innovative. Secondly, the impact of other adverse birth outcomes, such as premature rupture of membranes, placental abruption, and placenta praevia, has been barely studied in the limited studies on the co-morbidity of GDM and HDP, and the joint multiple outcome comparison study of these outcome indicators is also innovative. Thus, the current study aimed at further clarifying the effects of GDM and HDP co-morbidity on multiple adverse birth outcomes using data from the maternal and child health care system and the clinical examination information system of Zhoushan Maternal and Child Care Hospital in Zhoushan, Zhejiang province, China.

## 2 Materials and methods

### 2.1 Participants and study design

**Figure 1:**
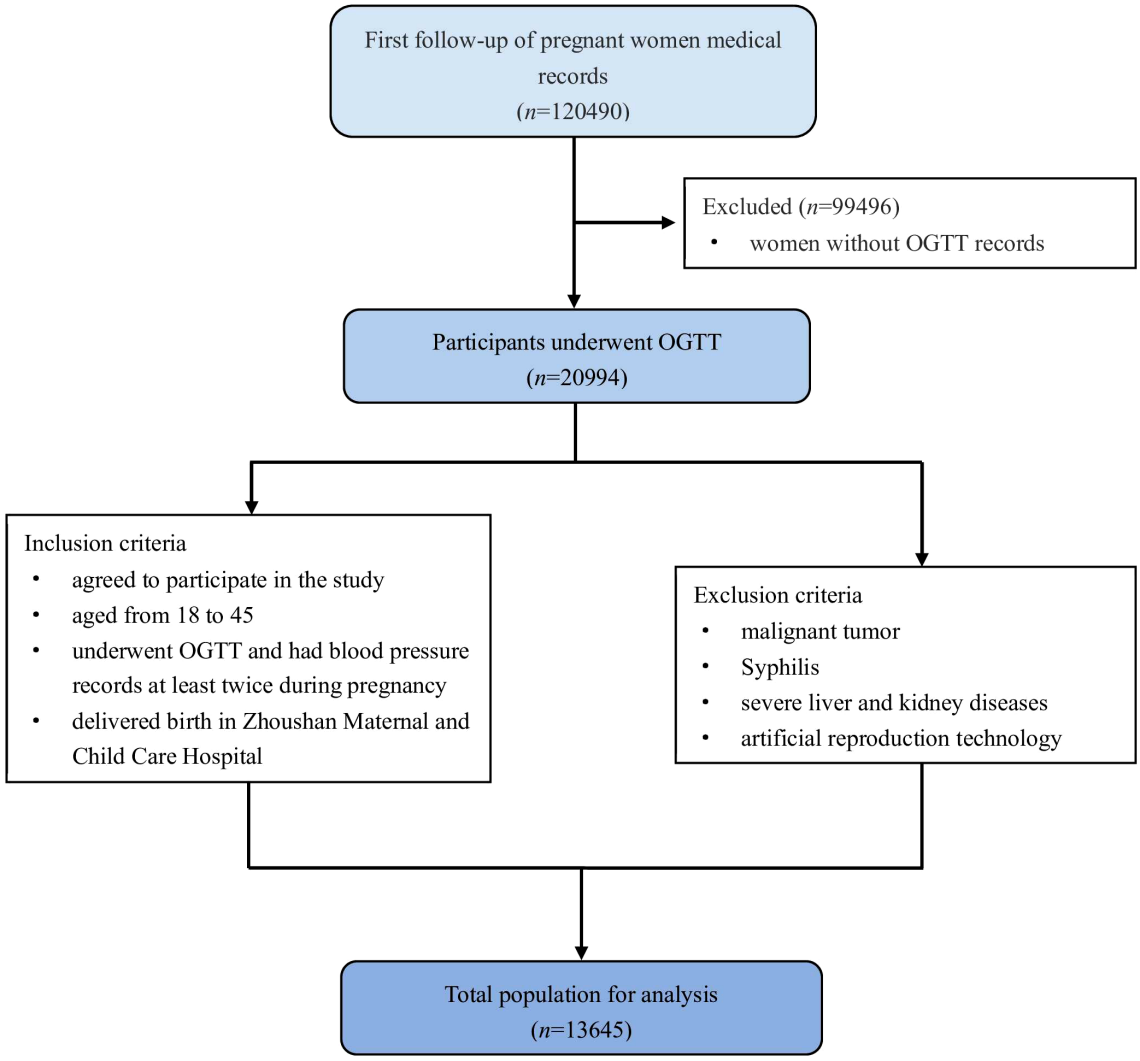
Flowchart of participants in the study.

This was a retrospective cohort study using data from the comprehensive electronic medical recorder system (EMRS) of Zhoushan Maternal and Child Care Hospital in Zhoushan, Zhejiang province, China, between 2015 and 2022. The study protocol was approved by the institutional review board of the School of Medicine in Zhejiang University. First follow-up information, oral glucose tolerance test (OGTT), prenatal follow-up blood pressure data, and delivery information were matched with unique IDs. The inclusion criteria were: (1) aged from 18 to 45; (2) underwent OGTT and had blood pressure records at least twice during pregnancy; (3) delivered birth in this hospital. The exclusion criteria were: (1) malignant tumor; (2) Syphilis; (3) severe liver and kidney diseases; (4) artificial reproduction technology. Informed consent was obtained from all individual participants included in the study. The flowchart of participants in the study was showed in **Figure 1**.

### 2.2 Measurement of OGTT and definition of GDM and GDM subtypes

GDM screening has become a routine examination among pregnant women in China. OGTT was conducted between the 24^th^ and 28^th^ week of gestation. After an overnight fast (at least 8 h), 75 g glucose resolved in 300 ml water was given and drunk within 5 min the next morning. Venous blood samples were taken at 0 h, 1 h, and 2 h during OGTT for measuring plasma glucose levels. Plasma glucose levels were immediately measured by the hexokinase method with commercially available kits (Beckman AU5800, Beckman Coulter Inc., Brea, CA, USA). The result of OGTT was extracted from EMRS, and GDM was diagnosed based on the criteria set by the IADPSG (13), those whose plasma glucose met at least one of the following criteria were diagnosed as GDM: fasting plasma glucose (FBG) ≥5.1 mmol/l; 1h-postprandial glucose (PG1H) ≥10 mmol/l; 2h-postprandial glucose (PG2H) ≥8.5 mmol/l. Thereby, three subgroups of GDM can be separated: isolated fasting hyperglycemia (IFH), isolated post-load hyperglycemia (IPH), and combined hyperglycemia (CH) according to OGTT results (14).

### 2.3 Measurement of blood pressure and definition of HDP

The measurements of SBP and DBP were taken as a part of routine perinatal care by physicians, the data of which were extracted from EMRS. BP measurements were performed in a seated position, from the right hand with a standard mercury sphygmomanometer. The gestational age was calculated by the last menstruation date and confirmed by the B ultrasound. HDP encompasses chronic hypertension, gestational hypertension, preeclampsia/eclampsia, and preeclampsia superimposed on chronic hypertension (15). Chronic hypertension was defined as having hypertension before pregnancy or diagnosed before 20 weeks gestation in at least two consecutive examinations (16). The gestational week of HDP onset was defined as the first gestational week of two consecutive examinations of diagnosing gestational hypertension. All participants were classified into three stages based on their gestational week of HDP onset, including ≤20^th^ week, 21-27^th^ week, and ≥28^th^ week.

### 2.4 Definition of delivery outcomes and covariables

There are two types of birth weight abnormalities according to the gestational age and sex-specific curves in the Chinese Journal of Pediatrics 2020: small for gestational age (SGA, birth weight<10^th^ percentile) and large for gestational age (LGA, birth weight>90^th^ percentile) (17). Preterm birth is defined as births before 37 completed weeks of gestation. Placenta previa is the complete or partial covering of the internal os of the cervix with the placenta (18). Pregnancy BMI was divided into four categories based on the Working Group on Obesity in China (19): underweight, BMI < 18.5 kg/m^2^; normal, BMI 18.5-23.9 kg/m^2^; overweight, BMI 24.0-27.9 kg/m^2^; obesity, BMI ≥ 28 kg/m^2^. GWG was calculated as the pre-natal weight minus pre-pregnancy weight.

### 2.5 Statistical analysis

The participants were divided into four groups: normal, GDM only, HDP only, and GDM+HDP (women with both GDM and HDP). The data were missing completely at random and deleted in rows. Continuous and categorical variables were respectively presented as mean ± SD and frequency (percentage). Analysis of variance (ANOVA) and Chi-square test were used for continuous variables and categorical variables, respectively, to compare the characteristics between groups. Multivariable linear regression model was used to analyze the associations of gestational age and birth weight with GDM and HDP. Multiple multivariable or multivariable logistic regression was used to estimate the adjusted odds ratio (aOR) with 95% confidence interval (CI) of each birth outcome measured for each group, relative to the comparison group. To control the confounding bias, the following variables were adjusted based on the distribution of characteristics and important demographic information: age, gravidity, history of 3 or more abortions, gestational age at OGTT, BMI at first visit, pregnancy season, and education. When the outcome variable was birth weight, delivery gestational age was also adjusted. To investigate whether the effects of GDM or HDP were independent of GWG, model 2 was further adjusted for GWG on model 1. All results were considered statistically significant at a value of *P* < 0.05. Statistical analyses were performed using R software (version 4.2.1) (http://www.R-project.org).

## 3 Results

There were 13645 pregnant women included in the analyses.10655 (78.1%) women were normal, 2341 (17.2%) women were diagnosed with GDM, 461 (3.4%) women were diagnosed with HDP, and 188 (1.3%) women were diagnosed with GDM and HDP. The maternal age, pre-pregnancy BMI, and weight gain during pregnancy were higher in the GDM+HDP group than in the other three groups, and the proportion of pregnancies with three or more, a history of abortion, and preterm were also higher than in the other three groups (**Table 1**). The average follow-up time was 27 weeks. The reason for missing follow-up might be pregnant women’s mobility.

**Table 1:** Comparison of basic characteristic in normal pregnant women, women with GDM alone, HDP alone, and both GDM and HDP.

Variable	Normal (N=10655)	GDM only (N=2341)	HDP only (N=461)	GDM+HDP (N=188)	*P*
Mean ± SD					
Age, years	28.58 ± 4.21	30.25 ± 4.40	29.82 ± 4.91	31.01 ± 5.39	<0.001
Gestational age at OGTT, week	25.83 ± 1.44	25.80 ± 1.44	25.97 ± 1.42	25.95 ± 1.48	0.093
BMI at first visit, kg/m^2^	21.06 ± 2.88	22.00 ± 3.26	23.57 ± 4.11	24.90 ± 3.97	<0.001
Gestational weight gain, kg	12.73 ± 5.64	10.66 ± 4.69	12.60 ± 4.80	9.52 ± 7.86	<0.001
N (%)					
Gravidity					0.001
1	4371 ± 41.0	859 ± 36.7	189 ± 41.0	68 ± 36.2	
2	2886 ± 27.1	634 ± 27.1	111 ± 24.1	47 ± 25.0	
≥3	3253 ± 30.5	820 ± 35.0	154 ± 33.4	72 ± 38.3	
Missing	145 ± 1.4	28 ± 1.2	7 ± 1.5	1 ± 0.5	
Education					<0.001
Junior high school and below	2127 ± 20.0	467 ± 19.9	112 ± 24.3	55 ± 29.3	
High school	1301 ± 12.2	307 ± 13.1	56 ± 12.1	24 ± 12.8	
College and above	4817 ± 45.2	909 ± 38.8	175 ± 38.0	55 ± 29.3	
Missing	2410 ± 22.6	658 ± 28.1	118 ± 25.6	54 ± 28.7	
History of abortion					0.043
No	10052 ± 94.3	2184 ± 93.3	429 ± 93.1	171 ± 91.0	
Yes	603 ± 5.7	157 ± 6.7	32 ± 6.9	17 ± 9.0	
History of preterm					0.002
No	10549 ± 99.0	2313 ± 98.8	451 ± 97.8	181 ± 96.3	
Yes	106 ± 1.0	28 ± 1.2	10 ± 2.2	7 ± 3.7	
Season of last menstruation					0.001
Spring	2039 ± 19.1	413 ± 17.6	115 ± 24.9	42 ± 22.3	
Summer	2507 ± 23.5	628 ± 26.8	107 ± 23.2	47 ± 25.0	
Autumn	3239 ± 30.4	723 ± 30.9	131 ± 28.4	60 ± 31.9	
Winner	2870 ± 26.9	577 ± 24.6	108 ± 23.4	39 ± 20.7	

HDP, hypertensive disorders complicating pregnancy; GDM, gestational diabetes mellitus; BMI, body mass index; OGTT, oral glucose tolerance test.

### 3.1 Association of GDM and HDP with gestational week of delivery and preterm birth

As shown in **Table 2**, GDM, HDP, and GDM+HDP were all inversely associated with the gestational week of delivery (β=-0.19, *P*<0.0001 for GDM; β=-0.49, *P*<0.0001 for HDP; β=-0.43, *P*=0.0022 for GDM+HDP). After further adjustment for GWG, the association of HDP (β=-0.54, *P*<0.0001) and GDM+HDP (β=-0.30, *P*=0.0291) with gestational week of delivery was consistent with that before adjustment, but no significant association was observed between GDM and gestational week of delivery. Thus, the gestational week of delivery was mainly influenced by HDP. However, While GDM and HDP (aOR=1.35, 95%CI: 1.08-1.66 for GDM; aOR=1.74, 95%CI: 1.15-2.53 for HDP) were positively associated with preterm birth, comorbidities were not significantly associated with preterm birth (**Supplementary Table S1**).

**Table 2:** The association of GDM and HDP with gestational week of delivery and fetal birth weight.

Group	N	Mean ± SD	Model 1^a^		Model 2^b^	
			β (se)	*P*	β (se)	*P*
Gestational week of delivery						
Normal	10655	38.99 ± 1.61	ref.	–	ref.	–
GDM only	2341	38.73 ± 2.42	-0.19(0.04)	<0.0001	-0.07(0.04)	0.2127
HDP only	461	38.42 ± 1.99	-0.49(0.09)	<0.0001	-0.54(0.09)	0.0002
GDM + HDP	188	38.43 ± 1.81	-0.43(0.14)	0.0022	-0.30(0.14)	0.0291
Birth weight ^c d^						
Normal	10562	3314.35 ± 451.64	ref.	–	ref.	–
GDM only	2317	3317.26 ± 503.71	3.82(9.83)	0.6977	39.65(9.69)	<0.0001
HDP only	458	3187.61 ± 571.81	-109.49(20.80)	<0.0001	-126.52(20.27)	<0.0001
GDM + HDP	185	3265.43 ± 605.96	-66.01(32.16)	0.0401	-23.03(31.37)	0.4627

a Model 1 was adjusted for age, gravidity, history of 3 or more abortions, gestational age at OGTT, BMI at first visit, pregnancy season and education.

b Model 2 was adjusted variable1s in Model 1 and further adjusted for gestational weight gain.

c Model 1 was adjusted for age, delivery gestational age, gravidity, history of 3 or more abortions, gestational age at OGTT, BMI at first visit, pregnancy season and education.

d Model 2 was adjusted variable1s in Model 1 and further adjusted for gestational weight gain.

HDP, hypertensive disorders complicating pregnancy; GDM, gestational diabetes mellitus.

### 3.2 Association of GDM and HDP with composite adverse birth outcomes

As shown in **Supplementary Table S2**, GDM was associated with an increased risk of Neonatal Jaundice (aOR=2.21, 95%CI: 1.05-4.40), and HDP was associated with an increased risk of preterm birth (aOR=1.87, 95%CI:1.24-2.74) and premature rupture of membranes (aOR=0.66, 95%CI: 0.46-0.93) in model 2. Considering that GDM and HDP have different effects on outcomes, we further selected preterm birth, placenta praevia, and neonatal jaundice as composite outcomes (**Table 3**). After adjustment for confounders, GDM, HDP, and GDM+HDP were all positively associated with the risk of composite outcomes (aOR=1.35, 95%CI: 1.10-1.66 for GDM; aOR=1.78, 95%CI: 1.20-2.55 for HDP; aOR=1.82, 95%CI: 1.02-3.04 for GDM+HDP). In Model 2, only HDP was positively associated with the risk of composite outcomes (aOR=1.89, 95%CI: 1.28-2.73), whereas GDM and GDM+HDP were not significantly associated with the risk of composite outcomes (**Table 3**).

**Table 3:** The association of GDM and HDP with adverse birth outcomes.

Group	Composite pregnancy outcomes^*^		SGA (< 10th centile)		LGA (> 90th centile)	
	Model 1^a^ aOR (95% CI)	Model 2^b^ aOR (95% CI)	Model 1^a^	Model 2^b^	Model 1^a^	Model 2^b^
			aOR (95% CI)	aOR (95% CI)	aOR (95% CI)	aOR (95% CI)
Normal	ref	–	ref.	–	ref.	–
GDM* only	1.35 (1.10-1.66)	1.16 (0.93-1.42)	1.07 (0.88-1.3)	0.97 (0.79-1.19)	1.38 (1.21-1.56)	1.29 (1.12-1.48)
HDP* only	1.78 (1.20-2.55)	1.89 (1.28-2.73)	2.19 (1.57-3.06)	2.29 (1.64-3.19)	0.98 (0.74-1.3)	0.68 (0.5-0.92)
GDM + HDP	1.82 (1.02-3.04)	1.43 (0.79-2.45)	2.2 (1.24-3.92)	1.93 (1.07-3.46)	2.33 (1.64-3.31)	1.64 (1.12-2.41)

^*^ Composite pregnancy outcomes = At least one of Preterm birth, Placenta previa and Neonatal Jaundice.

a Model 1 was adjusted for age, gravidity, history of 3 or more abortions, gestational age at OGTT, BMI at first visit, pregnancy season and education.

b Model 2 was adjusted variable1s in Model 1 and further adjusted for gestational weight gain.

HDP, hypertensive disorders complicating pregnancy; GDM, gestational diabetes mellitus; aOR, adjusted odds ratio; CI: confidence interval; AGA, appropriate for gestational age; SGA, small for gestational age; LGA, large for gestational age.

### 3.3 Association of HDP and GDM with birth weight and SGA/LGA

As shown in **Table 2**, HDP and GDM+HDP groups were associated with decreased birth weight (β= -109.49, *P*<0.0001 for HDP; β= -66.01, *P*=0.0401 for GDM+HDP). In model 2, GDM was associated with increased birth weight (β= 39.65, *P*<0.0001), but no significant association was observed between GDM+HDP and birth weight. The association between HDP and birth weight was consistent with that before adjustment (β= -126.52, *P*<0.0001).

As shown in **Table 3**, after adjustment for confounders, GDM was associated with an increased risk of delivering an LGA baby (aOR=1.38, 95%CI 1.21-1.56), while HDP was associated with an increased risk of delivering an SGA baby (aOR=2.19, 95%CI 1.57-3.06). Furthermore, GDM+HDP was associated with increased risk of both SGA (aOR=2.2, 95%CI 1.24-3.92) and LGA (aOR=2.33, 95%CI 1.64-3.31), and the associations were stronger than that in only GDM or HDP women. After adjusting for GWG in Model 2, the results were consistent with that before adjustment.

### 3.4 Association of the comorbidity of GDM and HDP diagnosed at different trimester with gestational week of delivery and birth weight

Considering that diagnosis of HDP at different periods may have different effects on outcomes, we further analyzed the associations of diagnosis of HDP combined with GDM at three periods on gestational weeks and birth weight. As shown in **Table 4**, HDP diagnosed at all periods were associated with decreased gestational week of delivery, and HDP diagnosed at 21-27th week have the strongest association (β = -0.96, P<0.001). Furthermore, after being combined with GDM, the association became even stronger (β = -1.57, P<0.001).

**Table 4:** The association of GDM and HDP diagnosed at different times with gestational week of delivery and birth weight.

Group		N	Mean ± SD	Model 1^a^		Model 2^b^	
				β (se)	*P*	β (se)	*P*
GDM	HDP	Gestational week of delivery					
No	No	10562	38.98 ± 1.56	ref	–	ref	–
Yes	No	2317	38.75 ± 1.70	-0.05 (0.04)	0.1931	-0.16 (0.04)	<0.0001
No	≤20th week	130	38.28 ± 2.07	-0.53 (0.14)	0.0002	-0.55 (0.15)	0.0002
No	21-27th week	52	38.29 ± 1.85	-0.96 (0.24)	0.0001	-1.05 (0.25)	<0.0001
No	≥28th week	43	37.88 ± 3.20	-0.48 (0.10)	<0.0001	-0.40 (0.10)	0.0001
Yes	≤20th week	18	37.22 ± 3.19	-0.15 (0.22)	0.5033	-0.41 (0.23)	0.0702
Yes	21-27th week	285	38.53 ± 1.63	-1.57 (0.43)	0.0002	-1.73 (0.43)	0.0001
Yes	≥28th week	115	38.63 ± 1.35	-0.26 (0.16)	0.1003	-0.33 (0.16)	0.0366
GDM	HDP	Birth weight ^cd^					
No	No	10562	3314.35 ± 451.64	ref.	–	ref.	–
Yes	No	2317	3317.26 ± 503.71	4.63 (9.44)	0.6238	36.79 (9.33)	0.0001
No	≤20th week	130	3185.54 ± 563.15	-139.63 (42.05)	0.0009	-137.25 (41.08)	0.0008
No	21-27th week	43	3053.49 ± 782.53	-105.85 (45.59)	0.0203	-97.99 (44.53)	0.0278
No	≥28th week	285	3208.79 ± 536.98	-66.14 (25.26)	0.0088	-96.23 (24.71)	0.0001
Yes	≤20th week	52	3200.58 ± 570.57	-130.45 (64.35)	0.0426	-70.51 (62.90)	0.2624
Yes	21-27th week	18	3025.88 ± 973.00	-189.57 (76.97)	0.0138	-110.74 (75.26)	0.1412
Yes	≥28th week	115	3330.17 ± 544.26	19.31 (38.79)	0.6186	39.42 (37.90)	0.2983

a Model 1 was adjusted for age, gravidity, history of 3 or more abortions, gestational age at OGTT, BMI at first visit, pregnancy season and education.

b Model 2 was adjusted variable1s in Model 1 and further adjusted for gestational weight gain.

c Model 1 was adjusted for age, delivery gestational age, gravidity, history of 3 or more abortions, gestational age at OGTT, BMI at first visit, pregnancy season and education.

d Model 2 was adjusted variable1s in Model 1 and further adjusted for gestational weight gain.

HDP, hypertensive disorders complicating pregnancy; GDM, gestational diabetes mellitus.

Without GDM, HDP diagnosed before the 20^th^ week was most strongly associated with decreased birth weight (β = -139.63, *P*<0.001), but after being combined with GDM the association was reduced (β = -130.45, *P*=0.0426). However, GDM combined with HDP diagnosed at 21-27^th^ week was most strongly associated with decreased birth weight in each group (β = -189.57, *P*=0.0138) (**Table 4**). After additional adjustment for GWG in Model 2, the results were consistent with those before the adjustment.

### 3.5 Association of the comorbidity of HDP and GDM subtypes with gestational week of delivery and birth weight

GDM subtypes might also have different effects on outcomes, we further analyzed the associations of three GDM subtypes combined with HDP with gestational week of delivery and birth weight. As shown in **Table 5**, IPH (β = -0.12, *P*=0.0239) and CoH (β = -0.82, *P*<0.0001) without HDP were associated with decreased gestational weeks. After being combined with HDP, the association became stronger (β = -0.53, *P*=0.0100 for HDP+IPH; β= -0.83, *P*=0.0021 for HDP+CH). In addition, only CH (β = 87.87, *P*=0.0003) was associated with increased birth weight. However, after being combined with HDP, the association became non-significant, while IPH combined with HDP is associated with decreased birth weight (β= -129.67, *P*=0.0054) (**Table 4**). After additional adjustment for GWG in Model 2, CH was associated with increased birth weight (β = 159.42, *P*=0.0080).

**Table 5:** The association of GDM subtypes and HDP with gestational week and birth weight.

Group		N	Mean ± SD	Model 1^a^		Model 2^b^	
				β (se)	*P*	β (se)	*P*
HDP	GDM		Gestational week of delivery				
No	No	10655	38.99 ± 1.61	ref.	–	ref.	–
Yes	IFH	582	39.02 ± 1.46	-0.03 (0.08)	0.7180	0.01 (0.08)	0.9277
No	IPH	1440	38.75 ± 1.71	-0.12 (0.05)	0.0239	0.01 (0.05)	0.8574
No	CH	319	38.14 ± 5.03	-0.82 (0.11)	<0.0001	-0.65 (0.11)	<0.0001
No	No	461	38.42 ± 1.99	-0.50 (0.09)	<0.0001	-0.54 (0.09)	<0.0001
Yes	IFH	50	39.02 ± 1.79	0.07 (0.26)	0.8024	0.17 (0.26)	0.5190
Yes	IPH	88	38.32 ± 1.82	-0.53 (0.20)	0.0100	-0.45 (0.20)	0.0252
Yes	CH	50	38.04 ± 1.68	-0.83 (0.27)	0.0021	-0.55 (0.27)	0.0388
HDP	GDM		Birth weight ^cd^				
No	No	10655	3315.09 ± 452.43	ref.	–	ref.	–
No	IFH	582	3384.22 ± 484.15	14.41 (18.62)	0.4389	24.79 (18.15)	0.1719
No	IPH	1440	3281.43 ± 485.99	-17.32 (11.94)	0.147	25.31 (11.75)	0.0313
No	CH	319	3361.56 ± 586.49	87.87 (24.37)	0.0003	140.43 (23.81)	<0.0001
Yes	No	461	3187.77 ± 570.68	-107.67 (20.79)	<0.0001	-124.78 (20.25)	<0.0001
Yes	IFH	50	3321.84 ± 559.93	-80.04 (60.75)	0.1877	-43.40 (59.15)	0.4631
Yes	IPH	88	3179.77 ± 629.50	-129.67 (46.63)	0.0054	-108.36 (45.40)	0.0170
Yes	CH	50	3358.78 ± 592.65	69.97 (61.60)	0.2561	159.42 (60.06)	0.0080

a Model 1 was adjusted for age, gravidity, history of 3 or more abortions, gestational age at OGTT, BMI at first visit, pregnancy season and education.

b Model 2 was adjusted variable1s in Model 1 and further adjusted for gestational weight gain.

c Model 1 was adjusted for age, delivery gestational age, gravidity, history of 3 or more abortions, gestational age at OGTT, BMI at first visit, pregnancy season and education.

d Model 2 was adjusted variable1s in Model 1 and further adjusted for gestational weight gain.

HDP, hypertensive disorders complicating pregnancy; GDM, gestational diabetes mellitus; IFH, isolated fasting hyperglycemia; IPH, isolated post hyperglycemia; CH, Combined fast and post hyperglycemia.

## 4 Discussion

Our study demonstrated that women with both GDM and HDP had a higher risk of adverse neonatal outcomes than those with one comorbidity. This might be due to the severity, subtype, and time of diagnosis of GDM or HDP. Further analysis showed that women with GDM and HDP diagnosed in the 21-27^th^ week had the lowest gestational age at delivery and birth weight. As for GDM subtypes, GDM-CH+HDP had the greatest association with gestational age at delivery while IPH combined with HDP was associated with decreased birth weight.

It is well established that women with HDP or GDM alone had higher risks of adverse birth outcomes. However, there were only several studies focused on the association with adverse outcomes in women with both comorbidities. Stella et al. (6)reported that, in a Cincinnati cohort based on clinical data of 14,480 women, the rates of LGA were significantly increased in women with combined diagnoses of GDM and gestational hypertension (GH), with an OR of 1.51 (95%CI 1.14-1.98). The study did not include chronic hypertension, preeclampsia, and eclampsia, and did not analyze the risk of both LGA and SGA. Our study found GDM+HDP group had a higher risk in both LGA and SGA, which may due to the severity of HDP or GDM in women with both diseases was not homogenous.

The effect of GDM and HDP in pregnant women varies among populations. A Taiwan cohort study based on 19,442 women (12) showed the women with HDP and GDM had higher risk of adverse neonatal outcomes, compared with those without HDP, with an OR of 4.84 (95%CI 4.34–5.40) for preterm delivery, an OR of 1.90 (95%CI 1.76–2.06) for Jaundice, an OR of 31.7 (95% CI 16.5-60.9) for LGA and an OR of 6.57 (95% CI 5.56-7.75) for SGA. The risk of the outcomes above in our study population might not be higher than in other populations. However, information like BMI was unavailable to adjust for these potential confounders in data analyses and did not establish a group of GDM, therefore the impact associated with GDM alone or with the severity of GDM could not be evaluated in this study. Another recent Chinese cohort of 1398 women with twin pregnancies (20) showed, that no associations were found between HDP alone and adverse neonatal outcomes in monochorionic (MC) twin neonates, whereas MC twins born to women with both GDM and HDP had longer gestational age, heavier birthweight and lower preterm birth risk, which might suggest that neonatal outcomes in twins were more affected by GDM than by HDP. The difference between our results and that might be due to the molecular mechanism and severity of GDM and HDP varied in twin and singleton pregnancy.

The impact of GDM and HDP on neonatal outcomes can be affected by the gestational age of HDP diagnosis and GDM subtypes. A London cross-sectional study reported that earlier onset of hypertension in the mother was positively associated with poorer fetal outcome in the third trimester (28 to 42 weeks of gestation), including gestational age at delivery, birth weight, and Apgar score at 1 min and 5 min (21). However, the study did not access hypertension onset before 28 weeks of gestation We further investigated women with HDP diagnosed at the 21-27^th^ week had the lowest gestational age at delivery, and HDP diagnosed before the 20^th^ week had the lowest birth weight.

The magnitude and significance of adverse pregnancy outcomes differed by various combinations of abnormal OGTT glucose values. A retrospective American report indicated while women with elevated post-load glucose concentrations were at higher risk for hypertension, preterm delivery, or infants with hyperbilirubinemia, women with elevated fasting glucose showed an increased risk of having LGA offspring (22). In addition, Wang et al. (23) observed that the incidence of LGA/macrosomia in women with elevated fasting and post-load glucose concentrations (GDM-CH) was significantly higher than that in women with normal fasting blood glucose and abnormal blood glucose (GDM-IPH); the incidence of GDM and adverse pregnancy outcomes in 4.4 mmol/L pregnant women is very low. The categorization based on abnormal OGTT values seems to provide a practicable basis for clinical risk stratification and future research. Our further investigation found that GDM-CH had the lowest birth weight and decreased gestational week of delivery, followed by GDM-IPH, whereas GDM-IFH was not significantly associated with these outcomes. However, the sample size of women with GDM-IFH was relatively small.

The mechanism for the association of maternal GDM and HDP with neonatal adverse outcomes has not been fully understood. Insulin resistance may be the first consideration as the reason for this association, because it was putative as the pathogenesis of HDP and GDM in pregnant women (24, 25), and cord plasma insulin correlated positively with birthweight and neonatal fat mass (26). We observed that women with both GDM and HDP have higher levels of BMI before pregnancy than the other groups, and BMI is a parameter that reflects insulin resistance. Therefore, the combination of GDM and HDP may imply more severe insulin resistance. Other systemic changes, such as inflammation, oxidative stress, and maternal vasculopathy also play an important part in the pathogenesis of GDM and HDP. Hyperglycemia-promoted inflammation and accumulation of reactive oxidative species (ROS) impair endothelial cells and eventually damage vascular function (27). When GDM is combined with HDP, accompanied by shallow placental implantation and even acute atherosclerosis of placental blood vessels (28), it results in further reduction of vessel dysfunction and placental perfusion, thereby limiting fetal growth and other adverse outcomes. However, it is reasonable to presume that in GDM, the placenta may overcompensate by increasing nutrient transport, even in cases of mild vascular dysfunction, which may lead to excessive fetal growth. Additionally, some studies found that GDM combined with HDP makes uterine smooth muscle more sensitive to oxytocin and more likely to induce preterm birth (29, 30). In short, the pathogenic changes result from a complex interplay between the placenta, the mother, and the fetus.

There were some strengths in the present study. Firstly, this retrospective cohort study evaluates broader birth outcomes in pregnant women with GDM/HDP alone and in those who have both GDM and HDP. Secondly, data on a variety of confounding variables, such as pregnancy season and education level, were collected and used in the final analysis. Thirdly, to the best of our knowledge, this was the first study to analyze birth outcomes in women with HDP in different pregnancies combined with GDM and women with different GDM subtypes combined with HDP. However, several limitations need to be acknowledged. Firstly, the sample size of women with GDM and HDP was relatively small, and the incidence of individual adverse outcomes was low, therefore the association with each outcome might not be accurately assessed. Secondly, our study was a single-center study, and the findings have reference value finiteness for populations in other regions. Multicenter studies should be conducted to clarify the impact of the co-existence of GDM and HDP on neonatal outcomes. Last, information on clinical interventions after diagnosis of GDM and HDP has not been collected, and interference of therapeutic factors cannot be excluded. Lifestyle is also an important confounding factor, but it is not acquired in our data either.

Pregnant women suffering from the comorbidity of GDM and HDP had a higher risk of adverse neonatal outcomes. To prevent poor perinatal outcomes, we recommend individualized management for pregnant women with comorbidities as following: (1) Early detection and monitoring; (2) Diet and lifestyle adjustments; (3) Preventive or therapeutic medications; (4) Frequent Fetal Monitoring; (5) Timely delivery planning. Future studies are needed to further evaluate the effects of the gestational age of HDP diagnosis, GDM subtypes, and their co-morbidities on birth outcomes.

## 5 Conclusion

In conclusion, pregnant women suffering from both GDM and HDP had a higher risk of adverse neonatal outcomes than those with one comorbidity. Meanwhile, the women with GDM and HDP diagnosed in the 21-27th week had the lowest gestational age at delivery and birth weight, and HDP+GDM-CH had the greatest association with gestational age. This suggested that high attention and active intervention should be paid to this situation.

## Data Availability

The raw data supporting the conclusions of this article will be made available by the authors, without undue reservation.
