# Supplementary material for: Association of the comorbidity of gestational diabetes mellitus and hypertension disorders of pregnancy with birth outcomes
Source: Front Endocrinol (Lausanne). 2024 Dec 12;15:1468820. doi: 10.3389/fendo.2024.1468820 (PMC11669500; doi:10.3389/fendo.2024.1468820)
Supplement: Supplementary file 1 [file Table1.docx]

Supplementary Material

# Supplementary Tables

| Table S1. The association of GDM and HDP with preterm delivery | | | |
| --- | --- | --- | --- |
| **Group** | **Preterm birth** | | |
|  | N (%) | **Model 1**^a^  aOR* (95% CI*) | **Model 2**^b^  aOR (95% CI) |
| **Normal** | 431(96.0) | ref. | ref. |
| **GDM* only** | 136(94.2) | 1.35(1.08-1.66) | 1.13(0.90-1.40) |
| **HDP* only** | 37(92.0) | 1.74(1.15-2.53) | 1.87(1.24-2.74) |
| **GDM + HDP** | 16(91.5) | 1.72(0.93-2.97) | 1.31(0.68-2.30) |
| ^a^ Model 1 was adjusted for age, gravidity, history of 3 or more abortions, gestational age at OGTT, BMI at first visit, pregnancy season, and education;  ^b^ Model 2 was adjusted variables in Model 1 and further adjusted for gestational weight gain.  HDP, hypertensive disorders complicating pregnancy; GDM, gestational diabetes mellitus; aOR, adjusted odds ratio; CI, confidence interval. | | | |

| Table S2. The association of GDM and HDP with pregnancy outcomes. | | | | |
| --- | --- | --- | --- | --- |
| **Outcome** | **Group** | N (%) | **Crude Model**  aOR (95% CI) | **Model 1**^a^  aOR (95% CI) |
| **Preterm birth** | Comparison | 431(69.5) | ref. | ref. |
|  | GDM only | 136(21.9) | 1.46(1.20-1.78) | 1.13(0.90-1.40) |
|  | HDP only | 37(6.0) | 2.07(1.44-2.90) | 1.87(1.24-2.74) |
|  | GDM + HDP | 16(2.6) | 2.21(1.26-3.60) | 1.31(0.68-2.30) |
| **Premature rupture of membranes** | Comparison | 1290(79.2) | ref. | ref. |
|  | GDM only | 284(17.4) | 1.00(0.87-1.15) | 1.00(0.86-1.16) |
|  | HDP only | 39(2.4) | 0.67(0.47-0.92) | 0.66(0.46-0.93) |
|  | GDM + HDP | 16(1.0) | 0.72(0.42-1.15) | 0.62(0.34-1.05) |
| **Fetal distress** | Comparison | 154(80.2) | ref. | ref. |
|  | GDM only | 28(14.6) | 0.82(0.54-1.22) | 0.95(0.60-1.46) |
|  | HDP only | 8(4.2) | 1.20(0.54-2.31) | 0.95(0.33-2.13) |
|  | GDM + HDP | 2(1.0) | 0.74(0.12-2.33) | 1.07(0.17-3.50) |
| **Placenta previa** | Comparison | 47(71.2) | ref. | ref. |
|  | GDM only | 14(21.2) | 1.36(0.72-2.40) | 1.01(0.49-1.93) |
|  | HDP only | 3(5.0) | 1.48(0.36-4.05) | 1.62(0.38-4.67) |
|  | GDM + HDP | 2(3.0) | 2.44(0.40-7.95) | 0.92(0.05-4.68) |
| **Neonatal Jaundice** | Comparison | 37(68.5) | ref. | ref. |
|  | GDM only | 12(22.2) | 1.50(0.75-2.79) | 2.21(1.05-4.40) |
|  | HDP only | 3(5.6) | 1.88(0.45-5.21) | 1.79(0.28-6.31) |
|  | GDM + HDP | 2(3.7) | 2.98(0.48-9.84) | 3.20(0.48-12.12) |
| ^a^ Model 1 was adjusted for age, gestational week of delivery, gravidity, education, history of preterm birth, history of 3 or more abortions, weight gain from first visit to pre-delivery, the season of last menstruation, and BMI at first visit.  HDP, hypertensive disorders complicating pregnancy; GDM, gestational diabetes mellitus; aOR, adjusted odds ratio; CI, confidence interval. | | | | |
